# Probing the Spin-Momentum Locking on Rashba Surfaces via Spin Current

**DOI:** 10.1021/acsami.4c06090

**Published:** 2024-07-16

**Authors:** José E. Abrão, Eudes Gomes da Silva, Gilberto Rodrigues-Junior, Joaquim B. S. Mendes, Antonio Azevedo

**Affiliations:** † Departamento de Física, Universidade Federal de Pernambuco, 50670-901 Recife, Pernambuco, Brazil; ‡ Departamento de Física, Universidade Federal de Viçosa, 36570-900 Viçosa, Minas Gerais, Brazil; § Department of Physics and Astronomy, University of Iowa, Iowa City, Iowa 52242, United States

**Keywords:** spintronics, Rashba surfaces, spin-momentum locking, spin-to-charge conversion, spin pumping

## Abstract

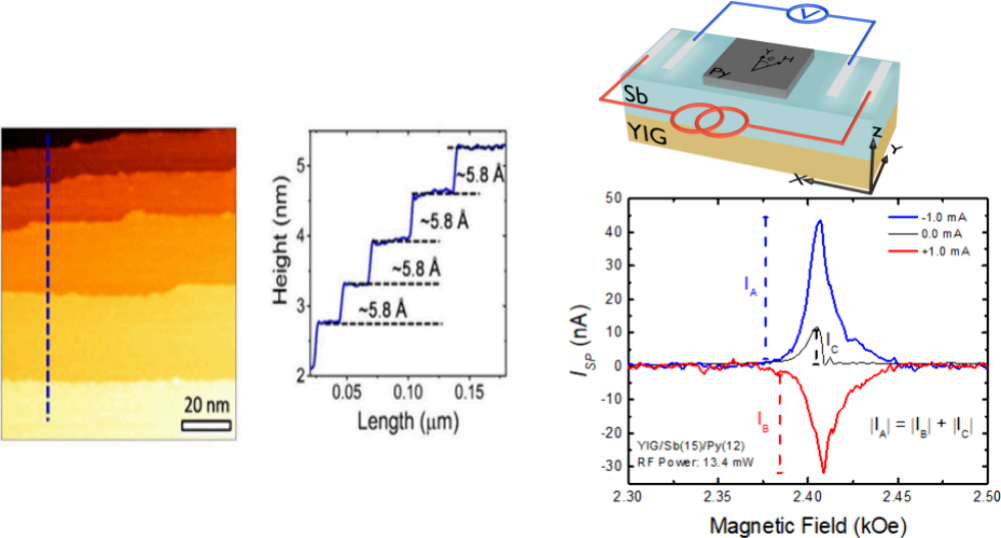

In this study, we investigate the spin-momentum locking phenomenon on Rashba states of antimony (Sb) films. Utilizing spin pumping in conjunction with an external charge current, we uncover the topological properties of Sb surface states. Our key finding is the precise manipulation of the direction and magnitude of the charge current generated by the inverse Rashba–Edelstein effect. This control is achieved through the dynamic interaction between out-of-equilibrium pumped spins and spin-momentum-locked flowing spins, which are perpendicular to the charge current. Our results highlight Sb as a promising material for both fundamental and applied spintronics research. The studied Sb nanostructures demonstrate potential for the development of low-power logic gates operating with currents in the microampere range, paving the way for advanced spintronic applications.

## Introduction

In the fascinating realm of condensed matter physics, spintronics has emerged as a groundbreaking field that exploits the spin of electrons for information processing and storage. Unlike traditional electronics, which relies solely on the charge of electrons, spintronics harnesses both the charge and spin of electrons, providing a new pathway for creating more efficient and versatile electronic devices.^1−3^ At the core of spintronics lies the fundamental concept of spin currents: currents that represent the flow of spin angular momentum. These currents can be used to transport information and to exert torques on magnetic materials, as well as converting the spin current into a charge current and vice versa.^4^

One prominent mechanism used to describe the spin-to-charge conversion in bulk materials is the direct and inverse spin Hall effect.^5^ The direct spin Hall effect (SHE) is a phenomenon where an applied electric field induces the generation of a perpendicular pure spin current to its direction. Unlike the conventional Hall effect, which involves the separation of charge carriers based on their charge, the direct spin Hall effect separates the charge carriers based on their spin states. The SHE is a consequence of spin–orbit coupling, an interaction between the spin of electrons and their motion in a crystal lattice with structural asymmetry^5^ or with a scattering center such as impurities or crystal defects.^6,7^ Conversely, a reciprocal effect entitled the inverse spin Hall effect (ISHE) exists.^8,9^ The ISHE is a phenomenon where a pure spin current, injected into a material exhibiting strong spin–orbit coupling, induces a charge accumulation perpendicular to both the spin polarization σ̂ and the injected spin current direction *J⃗*_S_. The charge current generated by the ISHE is described by
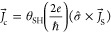
1where *θ*_SH_ is the spin Hall angle that represents the efficiency of the spin-to-charge conversion process, *ℏ* denotes the reduced Planck constant, and *e* is the elementary charge. The electrical voltage arising from ISHE-induced charge accumulation provides an experimental manner to translate spin information into a conventional electrical signal. As a fundamental mechanism in spintronics, ISHE holds promise for the development of memory, logic, and sensing devices. Moreover, it serves as a valuable tool for probing the physical properties of materials.^10,11^

**Figure 1:**
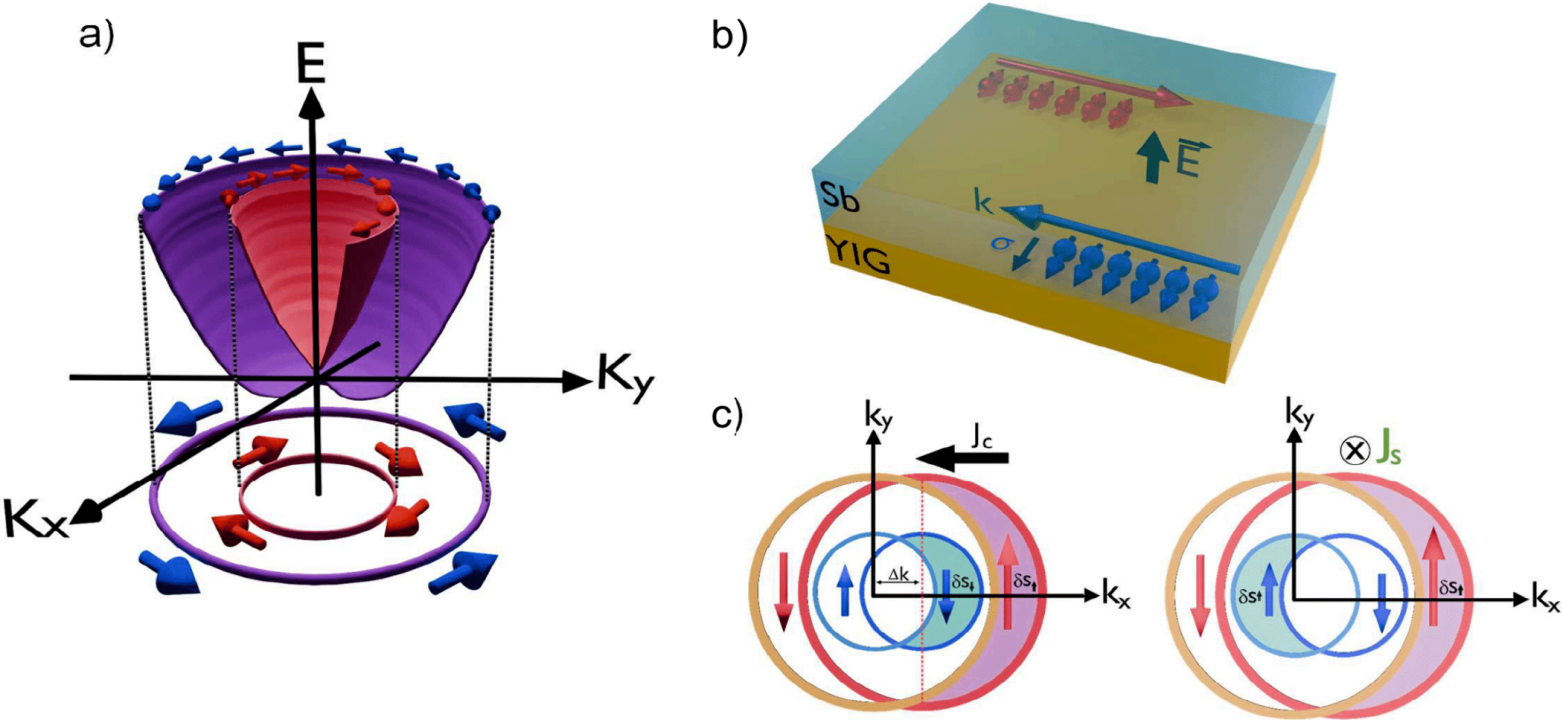
Schematics of the Rashba–Edelstein effects: (a) the energy splitting due to the Rashba spin–orbit coupling; it is possible to observe spin-momentum locking, where electrons with different spins travel through opposite directions. (b) A schematic representation of spin-momentum locking, characteristic of surface states, in the YIG/Sb layer. (c) The direct and inverse Rashba–Edelstein effects in a 2D electron gas. In direct REE, an electric field exerts a force on the electrons, resulting in a displacement of the Fermi circles. This displacement produces a nonequilibrium spin accumulation in a perpendicular direction. In inverse REE, a spin current pumped into the interface creates a spin accumulation that induces the displacement of the Fermi circles, resulting in a charge current in a perpendicular direction.

While SHE and ISHE are appropriate for bulk materials, surfaces and interfaces require consideration of additional significant effects: the direct and inverse Rashba–Edelstein effects. The direct Rashba–Edelstein effect (REE) emerges when electrons move through a plane that lacks structural inversion symmetry and simultaneously exhibits spin–orbit coupling. This effect can be understood as the manifestation of an effective magnetic field, even in the absence of an external magnetic field, on electrons in motion in their own reference frame.^12−15^ The effective magnetic field then couples to an electron’s magnetic moment through a Zeeman-like energy *μ*_B_*σ⃗* ·*B⃗*, where *σ⃗* and *μ*_B_ represent the vector of Pauli matrices and the Bohr magneton, respectively. Thereby, a steady nonequilibrium spin polarization with opposite sign on opposite edges of the sample is created.^16^ The hallmark of the Rashba–Edelstein effect is the formation of Rashba spin–orbit splitting, where electrons with opposite spins have different energies and momenta, causing them to move in opposite directions. This property is known as spin-momentum locking, and it is represented in Figure 1a. For surfaces and interfaces, the inversion symmetry is broken due to the absence/change of neighboring atoms, which produces an electric field, as represented in Figure 1b. It is worth mentioning that in recent works on effects in 2D gases with strong spin–orbit coupling, the theory of the Rashba–Edelstein effect is developed based on quantum kinetic equations and diagrammatic calculations for systems in which the spin–orbit interaction is linear in *k⃗*.^17−19^

Like the SHE/ISHE effects, a reciprocal effect of the direct Rashba–Edelstein effect, namely the inverse Rashba–Edelstein effect (IREE), exists. In the inverse effect, a nonequilibrium spin density *S⃗*_neq_, which is normally induced into the system by the injection of a pure spin current, induces a charge accumulation perpendicular to both *S⃗*_neq_ and the *ẑ*-direction of the symmetry breaking field. The charge current generated by the IREE is described by^20,21^
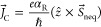
2A hallmark of the inverse Rashba–Edelstein effect is that the charge current converted by this effect does not depend on the direction of the spin current injected into the system; it depends only on the spin polarization σ̂ , which dictates the direction of *S⃗*_neq_ and *ẑ* represents the unit vector normal to the interface.

The direct and inverse Rashba–Edelstein effects have been explored in different systems such as graphene, transition metal dichalcogenides (TMDs), LaAlO_3_/SrTiO_3_, Bi/Ag and nGaAs/InGaAsP interfaces, and in topological insulators.^22−31^ Among materials with surface states, antimony (Sb) has proven to be a unique material. Sb is not only an elemental material but also a constituent element of several topological materials, and it can be a topological insulator by itself.^32,33^ Furthermore, a recent study showed that Sb films can be grown by sputtering without the need of heat treatment or the use of special substrates and still present surface states.^34^ Thus, Sb is a material that presents unique possibilities for different experiments and applications.

In a recent work, we explored the spin-to-charge conversion phenomenon in Co/Sb/Py heterostructure.^34^ Our findings revealed that the spin-to-charge conversion in Sb thin films was not dependent on the direction of the injected spin current, even for thicker films. Thus, no ISHE was observed in this material. Instead, the measured signal could only be attributed to IREE occurring at the Co/Sb and Sb/Py interfaces. To further explore antimony properties, in this current work we perform spin pumping measurements while applying an external charge current. The samples consisted of YIG/Sb(15 nm)/Py(12 nm) and YIG/Sb(15 nm)/Ti(3 nm) heterostructures, where YIG and Py stand for yttrium iron garnet (Y_3_Fe_5_O_12_) and permalloy (Ni_81_Fe_19_), respectively. The YIG films were produced by means of liquid phase epitaxy (LPE) following the traditional PbO/B_2_O_3_ flux method,^35^ while the Sb, Ti, and Py films were deposited by DC sputtering. The structural properties, surface roughness, and morphology of the sputtered grown Sb thin film are depicted in Figures S1–S3 in the Supporting Information of this manuscript. X-ray diffraction patterns confirm the crystalline and low surface roughness of the films, with preferential growth along the *c*-axis (see the Supporting Information for more details about the conditions of growth and the crystallographic structure of the Sb films).

## Results and Discussion

**Figure 2:**
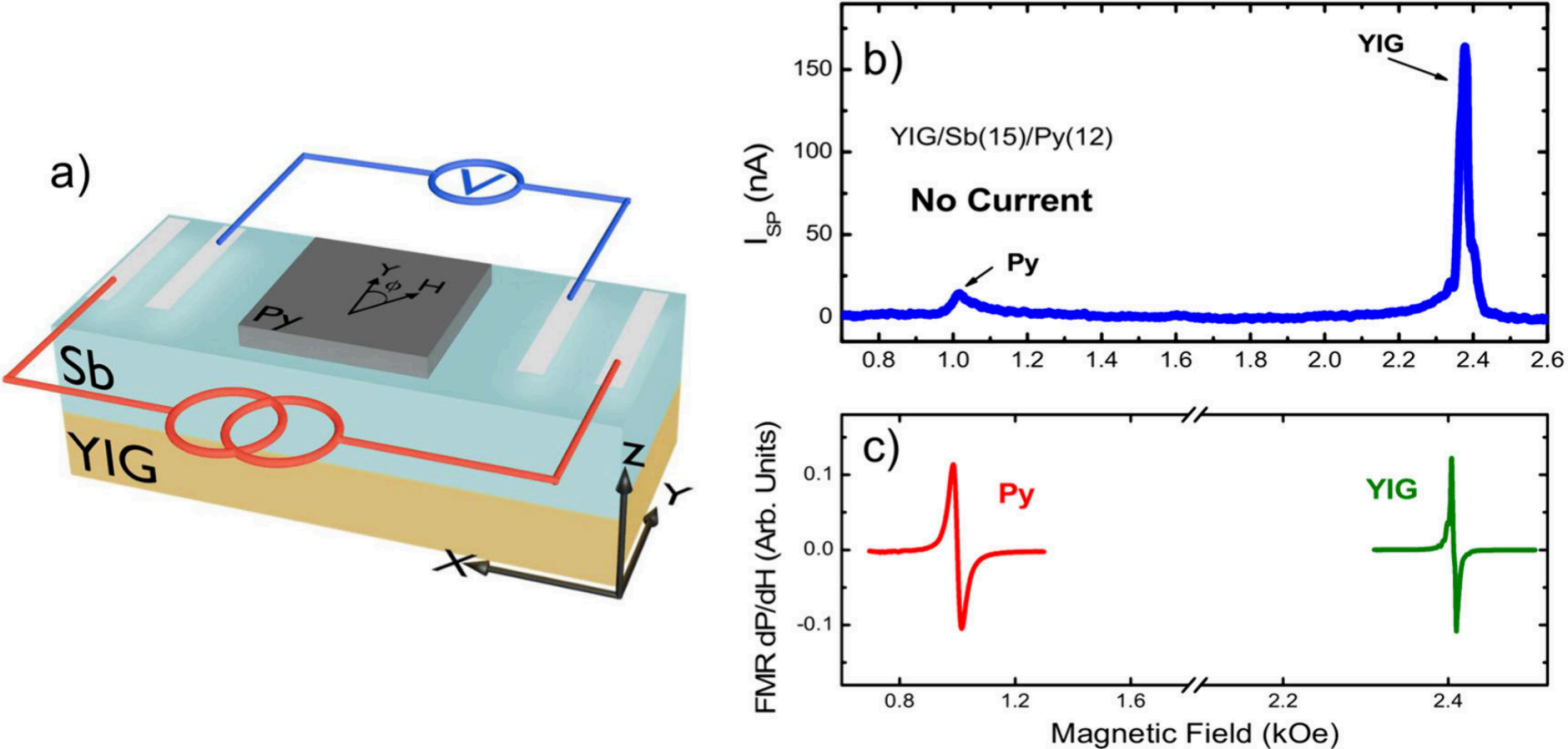
(a) Schematic of the experimental setup used for simultaneous measurements of spin pumping and DC current application, where electrodes were attached to the edges of the sample in a four-point configuration. (b) Spin pumping signal for YIG/Sb(15 nm)/Py(12 nm), with both signals displaying the same polarity. The applied RF power was 110 mW. (c) Derivative of the absorption FMR signal for both ferromagnets, presented separately due to the difference in microwave power needed to excite the FMR condition. To excite the FMR in Py and YIG, we used 110 and 0.01 mW, respectively.

The spin pumping, driven by ferromagnetic resonance (SP-FMR), measurements were made via a ferromagnetic resonance (FMR) spectrometer at room temperature, operating at 9.42 GHz. We mounted the sample on a PVC rod and placed it at the bottom of a rectangular microwave resonant cavity (TE_102_ mode) with a *Q*-factor of 2000, where the RF magnetic field is maximum, and the RF electric field is minimum. Four electrodes arranged in a four-point configuration (see Figure 2a) were then attached to the sample with silver paint. This configuration allows for the detection of the spin pumping signal while applying a charge current to the sample. Since YIG is an insulator, the flow of charge current will pass only through Sb, thus ensuring that any changes in the spin pumping signal due to the current applied to the film are exclusively attributed to the surface states. All the investigated samples were cut into sizes of 2 × 3 mm^2^.

The main objective of the initial experimental verification was to determine whether Sb, when grown onto YIG, maintains its surface states. To achieve this, spin pumping measurements were performed on YIG/Sb/Py trilayers with no applied current. If the system truly exhibits surface states and the spin-to-charge conversion is dominated by IREE, the spin pumping signal will not depend on the spin current direction. In this scenario, only the polarization of the spin current, which can be fixed by the external field, becomes significant. Consequently, the observation of two peaks with the same polarity is expected.^20^Figure 2 shows both the ferromagnetic resonance (Figure 2c) and the spin pumping signal for the YIG/Sb(15 nm)/Py(12 nm) trilayer (Figure 2b) with no current passing through the Sb layer. It is noteworthy that both signals are positive, indicating that the antimony grown on top of YIG indeed possesses surface states. In fact, the signature of topologically surface states on the sputtered grown Sb thin film can be corroborated through scanning tunneling spectroscopy measurements, as represented in Figure S4. A pertinent question is whether the bulk electronic states could contribute to the SP signal shown in Figure 2b. In a previous study, we investigated the dependence of the SP on the Sb layer thickness.^34^ Our findings indicated that the contribution of bulk states, if any, is negligible.

Furthermore, a subtle asymmetry is observed in the signal corresponding to the YIG resonance in Figure 2b,c. This asymmetry indicates an excessive use of RF power to drive the FMR condition, leading to the onset of nonlinear effects in the YIG. To mitigate these effects, it is necessary to reduce the microwave power used to excite the resonance. However, decreasing the RF power causes the Py absorption signal to not be detected on the same scale as the YIG signal. Consequently, we decided to disregard the Py signal and focus exclusively on the YIG resonance.

**Figure 3:**
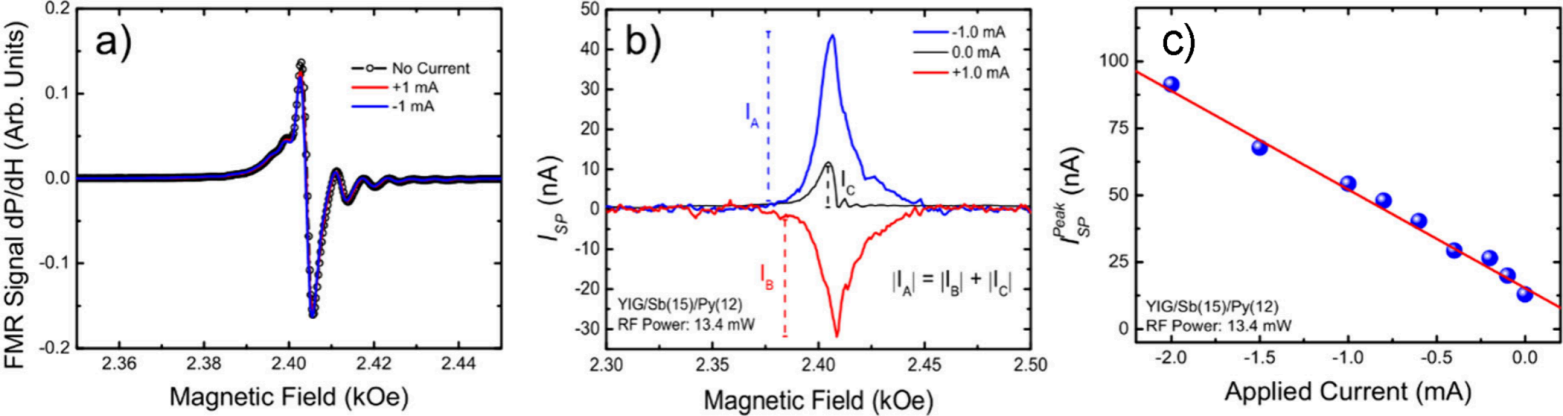
(a) Derivative of the FMR absorption curves for a typical a YIG/Sb(15 nm)/Py(12 nm) sample with ±1.0 mA and no applied current, confirming that in all cases, there is no change in the FMR field and line width. (b) Comparison of the spin pumping signal for ±1.0 mA and no applied current, highlighting the signal change polarities with respect to the applied current. (c) Linear dependency of the peak of the spin pumping signal as a function of the applied current.

After confirming that antimony grown onto YIG also exhibits surface states, measurements were conducted with an applied DC current. Initially, FMR measurements were performed with and without the external current, as shown in Figure 3a. Notably, no significant changes were observed, with the FMR resonance field and line width remaining constant at *H*_R_ = 2.415 kOe and Δ*H* = 2.4 Oe, respectively, across all resonance spectra. Due to the exceptional quality of our YIG film, magnetostatic modes are excited both below (surface modes) and above (volume modes) the FMR field. These modes are identified by the oscillations shown in Figure 3a. To fit the data, we used five Lorentzian functions: one for the surface mode, one for the FMR uniform mode, and three for the volume modes. The result indicates that the current flowing through the Sb layers does not introduce extra damping or change the FMR field. It is important to note that at the applied current value of 1.0 mA, the charge current density is insufficient to introduce any additional damping on the YIG magnetization by means of any spin transfer torque mechanism. Furthermore, the Oersted field generated by this DC current is negligible compared to the externally applied field of 2.405 kOe. Consequently, no change in the FMR field is observed. The spin pumping measurements presented in Figure 3b showed very different results. Thus, without any applied current, the signal for ϕ = 180° exhibits a single, well-defined peak pointing upward. Upon applying a current of −1.0 mA to the antimony layer, the measured signal increased by almost 4-fold. Interestingly, when inverting the applied current to +1.0 mA, not only does the signal become larger, but it also changes polarity. Moreover, numerical curve fitting revealed that the peak signal obtained for an applied current of −1.0 mA (*I*_A_) added to the signal for +1.0 mA (*I*_B_) is equivalent to the spin pumping signal without an external current (*I*_C_). To gain deeper insights into the impact of the applied current, a series of measurements were conducted by varying the current magnitude and polarity. The peak signal measured in the spin pumping experiment showed a linear behavior with the applied current, as shown in Figure 3c.

**Figure 4:**
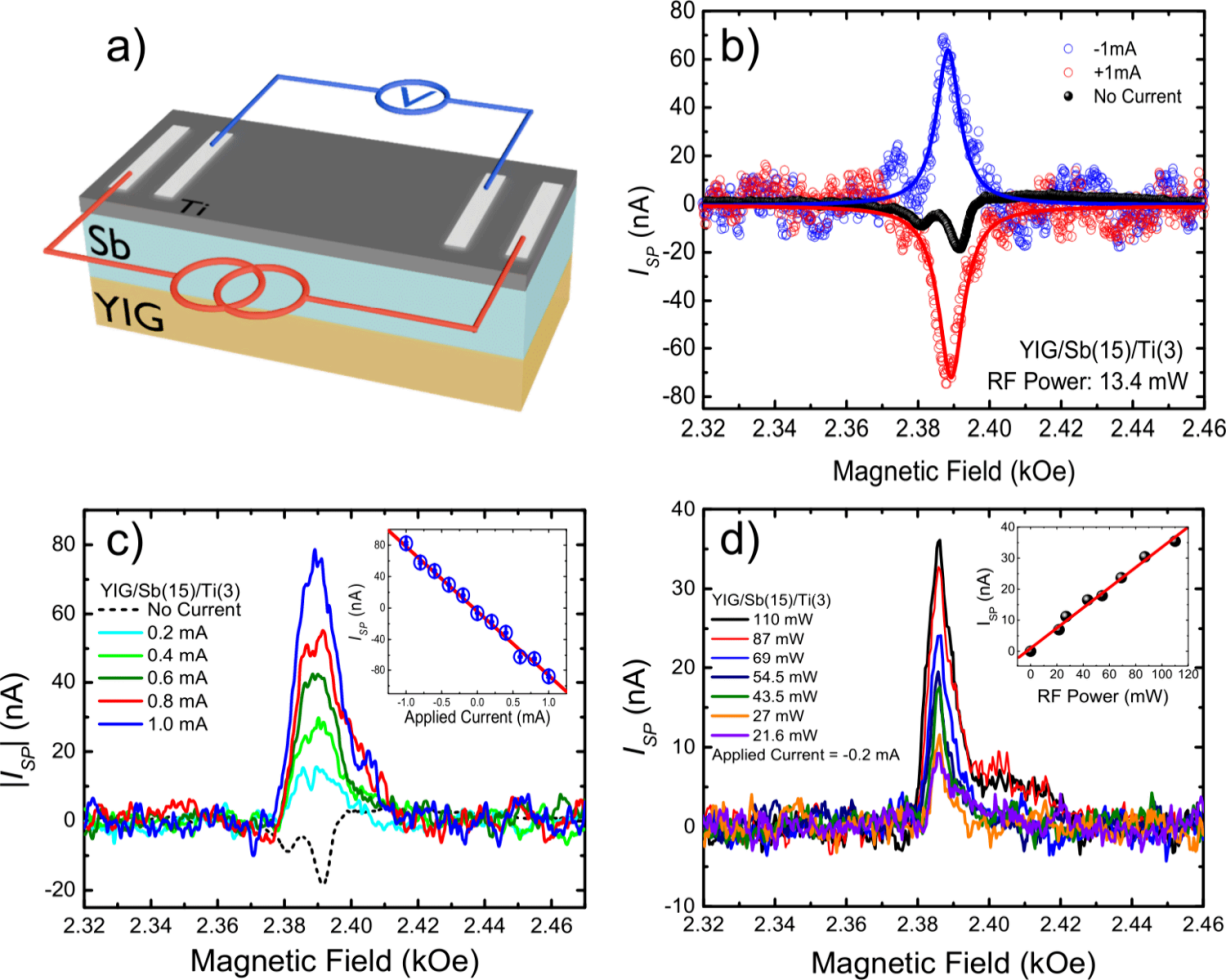
(a) Schematic illustration describing the measurement setup of SP-FMR for a typical YIG/Sb(15 nm)/Ti(3 nm) heterostructure. (b) Presentation of the spin pumping signal for ±1.0 mA of applied current and the signal with no applied current. Note that the signal reverses as the direction of the applied current changes. (c) Comparative analysis of the spin pumping signal for positive values of applied current. The inset shows the linear dependence of the peak spin pumping signal as a function of the applied current. (d) Comparative analysis of the spin pumping signal as a function of microwave power used to promote the FMR condition with an applied current of −0.2 mA passing through the sample. In the inset, the linear dependence of the measured signal as a function of the electromagnetic radiation power is presented.

The sample studied so far had a top layer of Py, as there was initially an interest in verifying whether antimony grown under YIG presented surface states. However, one might question whether Py influences the spin pumping measurement previously presented with applied current due to possible contamination of the measured signal via galvanomagnetic effects.^36,37^ To resolve this concern, YIG/Sb(15 nm)/Ti(3 nm) samples were prepared, where the Ti layer completely covers the Sb layer to protect it from natural oxidation. Figure 4 shows the results obtained by applying a current to the YIG/Sb(15 nm)/Ti(3 nm) sample. Notably, the results are similar to those previously observed. The application of current changes the measured signal drastically, being enhanced by more than 4 times just by applying a current of −1.0 mA. Moreover, when changing the polarity of the applied current from −1.0 mA to +1.0 mA, the signal also changes its polarity, as observed in Figure 4b. Based on these results, we can certainly conclude that the Py layer did not exert any influence on the system; instead, it merely served as a protective barrier to prevent oxidation. Moreover, it was also observed that the measured SP-FMR signal exhibited a linear relationship with the applied current, as shown in Figure 4c, where the inset shows the peak value as a function of the applied current. The linear fit indicates that a current of −41.7 μA is enough to compensate for the spin pumping signal. To further understand the system under study, measurements varying the microwave power while keeping the external applied current fixed at −0.2 mA were performed, and the results in Figure 4d show a comparison of the spin pumping signal for different values of microwave power used to promote the FMR condition. The numerical fit of each curve reveals that the peak signal behaves in a linear manner with the microwave power, as presented in the inset of Figure 4d.

The intriguing findings we observe can be explained by the interplay between the accumulation of nonequilibrium spins in Rashba states, arising from the combined effects of spin pumping and spin-momentum locking phenomena. In the Rashba–Edelstein effect, the flow of an electric current through a Rashba interface separates the electrons based on their spin states, naturally leading to a nonequilibrium spin density due to the energy difference between spin bands (see Figure 1a,c). During the spin pumping process, the magnetization dynamics at the YIG/Sb interface produce a spin accumulation that depends on the direction of the DC magnetic field and the RF power. This process induces a nonequilibrium spin density by shifting one of the Rashba surfaces at the Fermi level (see Figure 1c). Thus, introducing spin accumulation on a Rashba surface results in the emergence of a spatially uniform out-of-equilibrium spin density. This is manifested by changes in the Fermi surface radius for each spin state.^16,24^Figure 1c illustrates the direct and inverse Rashba–Edelstein effects, where the nonequilibrium spin density is represented by the difference in the colored areas for up and down spin densities. This dynamic interaction between the spin pumping spin accumulation and the spin-momentum-locked population is at the heart of our study and explains the observed phenomena.

If we consider a scenario in which a charge current *J⃗*_C_ is applied along the *x*-axis, it will result in a nonequilibrium spin density along the *y*-axis due to REE, denoted as *S⃗*_neq_^C^. At the same time, the spin pumping technique also induces a nonequilibrium spin density, represented by *S⃗*_neq_^SP^, at the interface. However, its direction depends on the direction of the external applied field, which pins the polarization of the spin current injected into the YIG/Sb interface. Depending on the relative orientation between *S⃗*_neq_^C^ and *S⃗*_neq_^SP^, the resulting spin density increases (when *S⃗*_neq_^C^and *S⃗*_neq_^SP^ are parallel) or decreases (when *S⃗*_neq_^C^and *S⃗*_neq_^SP^ are antiparallel). Thus, in the conducted experiments, there are two sources contributing to the resultant out-of-equilibrium spin density:
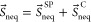
3The first contribution arises from the nonequilibrium spin polarization due to the injection of a spin current by SP-FMR into the Rashba states. In our experiment, this contribution depends on the experimental parameters related to the FMR, mainly the RF power. By varying the RF power, we observed a linear dependency, which is expected by due to the spin pumping effect (see Figure 4d).

The second contribution to the out-of-equilibrium spin density comes from the electric current applied to the system via the direct REE. This contribution varies with changes in the applied current, and notably, its spin polarization can invert with the reversal in the applied current. The contribution represented by the second term of eq 3 is always active whenever there is an electric current flowing. As we use the spin pumping effect to probe the spin polarity, which is locked perpendicular to the direction of the current flowing through the Sb layer, we only detect signal changes when the spin pumping current is being injected. The results presented in Figures 3 and 4 can be explained solely by the inverse Rashba–Edelstein effect in eq 2 if one considers that *S⃗*_neq_ contains a component dependent on the applied current passing through the Sb layer.

**Figure 5:**
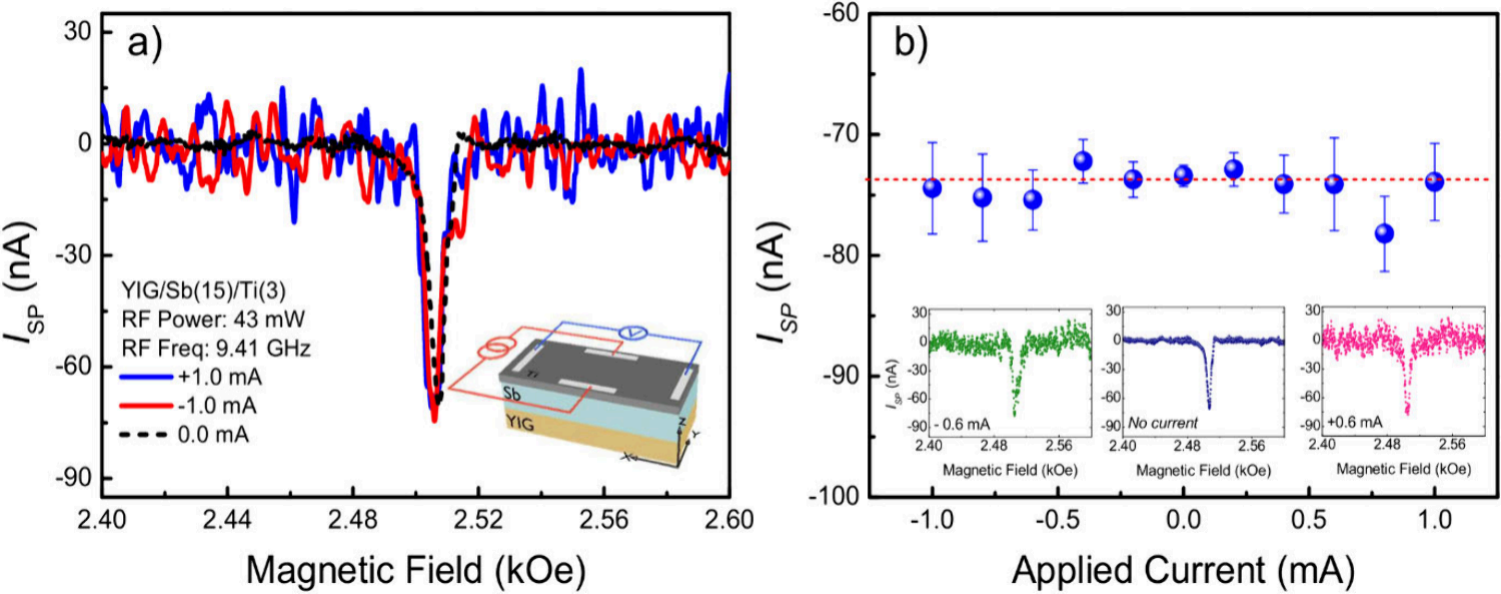
(a) A comparison of the spin pumping signal for an applied current of ±1.0 mA and no applied current,; the inset is a schematic of the experimental setup used in the second part of the experiments carried out in this work. (b) The peak of the spin pumping signal as a function of the applied current. In all cases investigated, it was verified that the *I*_SP_ signal remains constant.

To validate the hypothesis regarding the two terms that make up the nonequilibrium spin density, an experimental setup was developed in which now the current is applied perpendicular to the direction where we measured the spin pumping signal, represented in the inset of Figure 5a, similar to a planar Hall effect setup. In this new configuration, it is important to note that the out-of-equilibrium spin density due to REE is perpendicular to the direction of the applied current. Thus, when a current is applied in the *ŷ*-direction, the resulting out-of-equilibrium spin density is oriented along the *x̂*-direction. Consequently, it is anticipated that the electrical signal produced by the spin-momentum locking effect will not be detected due to the chosen measurement geometry. Figure 5a shows a comparison of the spin pumping signal, measured along *x̂*-direction, with an applied current of ±1.0 mA along the *ŷ*-direction. Notably, the SP-FMR signal does not change, meaning that the addition of current to the system only leads to an increase in the measurement noise, likely attributed to the thermoelectric nature of Sb. These results are consistent with the idea that the changes in the SP-FMR signal comes from a direct REE contribution to the out-of-equilibrium spin density. To validate these results comprehensively, measurements of SP-FMR with varying DC current intensity were performed. Figure 5b shows the peak value of SP-FMR for positive and negative values of the external applied current. In all cases, a peak value of approximately −75 nA was observed. This result once again reinforces the idea of a contribution to *S⃗*_neq_ due to the direct REE caused by the current passing through the sample. However, this extra component remains undetected due to the measurement geometry used.

## Conclusions

Our findings reveal that Sb grown onto YIG indeed possesses surface states, evidenced by the independence of the spin pumping signal on the injection direction. We further investigated the spin-to-charge conversion by introducing an external DC current into the system. Due to the spin-momentum locking, the nonequilibrium spin density produced in the experiment has two components: one originating from spin pumping and the other arising from the REE induced by the DC current. Thus, the measured signal is explained by
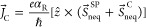
4Surprisingly, our investigation demonstrates the ability to manipulate the polarity and intensity of the spin-to-charge conversion by varying the applied charge current. In another way, we demonstrate the feasibility of interacting with a spin current using an electric current within a Rashba interface, paving the way for advanced spintronic applications.

## Experimental Section

### Sample Preparation

The sample preparation procedure involved two stages: the growth of the YIG films using liquid phase epitaxy and the growth of Sb films by magnetron sputtering. YIG (Y_3_Fe_5_O_12_) films were grown onto a 1 in. diameter commercial GGG (Gd_3_Ga_5_O_12_) substrate via liquid phase epitaxy. Fe_2_O_3_, Y_2_O_3_, B_2_O_3_, and PbO powders were weighed and placed into a platinum crucible, which was then inserted into a vertical tubular furnace. The furnace temperature was set to 940 °C with a rate of 1 °C/min. Once the furnace was up to temperature, the GGG substrate was then placed on a platinum sample holder that slowly descended into the crucible. To grow the YIG film, the GGG substrate dipped into the melt inside the crucible. To ensure a uniform film, the dipping process was done with the sample rotating at a fixed velocity. Once the YIG was grown, the film was cut into 3 × 2 mm^2^ samples with a low-speed diamond saw. Following this, the samples were cleaned in an ultrasonic bath with acetone and isopropyl alcohol. They were then placed inside the sputtering chamber, which was pumped down to a base pressure of 2.0 × 10^–7^ Torr or lower. The Sb films were grown by DC sputtering in an argon atmosphere with a working pressure of 2.7 × 10^–3^ Torr. The argon flux to the chamber was 550 sccm, and the plasma current was kept fixed at 50 mA.

### X-ray Diffraction

The crystal structure and interface quality of the sputtered Sb thin film on the (111) GGG substrate were investigate by means of X-ray diffraction (XRD) and X-ray reflectivity (XRR) methods using a four-circle high-resolution Bruker D8 Discover diffractometer equipped with a Cu K_α_ (λ = 1.5418 Å) radiation source and a two-bounce Ge (220) monochromator. The crystalline orientation of the Sb film was investigated via 2*θ–θ* XRD scans, performed in a high-resolution configuration (HRXRD), which indicated a highly *c*-axis oriented growth of Sb on the (111) GGG substrate. In order to probe crystal planes that were not necessarily aligned to the substrate surface as well as attenuate the diffracted signal associated with the GGG substrate, the overall crystalline structure of the sputtered Sb film was characterized through X-ray diffraction in a grazing incidence configuration (GIDXRD). Lastly, the film thickness and surface roughness were investigated by X-ray reflectivity measurements in the sputtered Sb thin films.

### Scanning Tunneling Microscopy (STM) and Spectroscopy (STS) Characterizations of Sb Films

Scanning tunneling microscopy (STM) and spectroscopy (STS) measurements were performed in order to investigate the surface electronic properties of the Sb thin film. In a typical STS curve, the differential tunneling conductance (d*I*/d*V*) is proportional to the local density of states (LDOS) at the STM tip position and, therefore, allows us to probe the presence of topologically protected states on the antimony surface. The STM and STS measurements were performed under ultrahigh vacuum conditions in an Omicron-VT STM system operating at room temperature with a base pressure of 1.0 × 10^–10^ mbar. All STM images were acquired using electrochemically etched polycrystalline tungsten (W) tips in constant current mode, and for STS measurements, a lock-in amplifier (operating at 3000 Hz) was used to obtain differential tunneling conductance (d*I*/d*V*) curves directly.

### Ferromagnetic Resonance (FMR)

Ferromagnetic Resonance (FMR) measurements were performed in a homemade spectrometer. The sample was mounted on top of a PVC rod and through a small hole in a rectangular microwave cavity operating at TE102 with a resonance frequency of 9.42 GHz. The cavity was then placed inside the poles of an electromagnet. The microwave frequency used to excite the FMR condition was directed into the sample via a circulator and wave guides that were connected to the cavity. On the third side of the circulator, an RF Schottky diode was placed, which allowed us to observe the reflected microwave power as a function of the applied external field. To have a better noise-to-signal ratio, the external field was modulated with two coils in the Helmholtz configuration, which were supplied with an AC signal of 1.1 kHz. The diode signal was then sent to a lock-in amplifier.

### Spin Pumping Measurements

Spin pumping measurements were performed in the same FMR spectrometer. The magnetization dynamics pumped a pure spin current from the ferromagnetic material into the adjacent layer. Inside the adjacent layer, the spin current could be converted into a charge current via the inverse Rashba effect or inverse spin Hall effect. The electrical signal produced at the resonance condition could be detected via a nanovoltmeter by attaching two electrodes at the edges of the sample with silver paste. To reduce the noise in the measurements, the external field modulation used to obtain the FMR spectra was turned off during the spin pumping measurements.

## Data Availability

The data that support the findings of this study are available from the corresponding authors upon reasonable request.

## Supporting Information Available

X-ray diffraction data of the Sb films used in this work, STM and STS characterizations of Sb films, sample fabrication details, and FMR and spin pumping measurements (PDF)
