# Supplementary material for: Probing the Spin-Momentum Locking on Rashba Surfaces via Spin Current
Source: ACS Appl Mater Interfaces. 2024 Jul 16;17(9):13162–9. doi: 10.1021/acsami.4c06090 (PMC11891834; doi:10.1021/acsami.4c06090)
Supplement: Supplementary file 1 — am4c06090_si_001.pdf [file am4c06090_si_001.pdf]

SUPPLEMENTARY INFORMATION

Probing the Spin-Momentum Locking in Rashba Surfaces via Spin Current

José E. Abrão[1,], Eudes Gomes da Silva[1,3], Gilberto Rodrigues-Junior[2], Joaquim B. S. Mendes[2,*], Antonio Azevedo[1,*]*

[1]Departamento de Física, Universidade Federal de Pernambuco, 50670-901 Recife, PE, Brazil.

[2]Departamento de Física, Universidade Federal de Viçosa, 36570-900 Viçosa, MG, Brazil.

[3]Department of Physics and Astronomy, University of Iowa, Iowa City, Iowa, USA.

Corresponding Authors

José E. Abrão – E-mail: elias.abrao@ufpe.br

Joaquim B. S. Mendes - E-mail: joaquim.mendes@ufv.br

Antonio Azevedo – E-mail: antonio.azevedo@ufpe.br

I. The X-ray diffraction of the Sb films used in this work

The crystal structure and interface quality of sputtered Sb thin film on (111) GGG substrate were investigate by means of x-ray diffraction (XRD) and x-ray reflectivity (XRR) method using a four-circle high-resolution Bruker D8- discover diffractometer equipped with Cu K_α (λ = 1.5418 Å) radiation source and a 2-bounce Ge (220) monochromator. Figure S1(a) shows a symmetrical (2θ-θ) XRD scan for the Sb thin film, performed in a high-resolution configuration (HRXRD). In addition to the peaks belonging to GGG substrate, it is possible to observe peaks that can be indexed with the Sb rhombohedral crystal structure with in-plane and out-of-plane lattice parameters of 4.30 Å and 11.22 Å, respectively.[1] Since in this configuration we are able to probe crystal planes that are oriented parallel to the substrate surface, the appearance of only (003n) diffraction peaks indicates a highly c-axis oriented growth of Sb on (111) GGG substrate. Indeed, the hexagonal atom arrangement characteristic of (111) planes in GGG cubic crystal can favor the preferential growth of layered structured materials along the out-of-plane direction without any thermal treatment which is crucial to preserve the magnetic properties of grown films.

In order to probe crystal planes that are not necessarily aligned to substrate surface as well as attenuate the diffracted signal associated with the GGG substrate, the overall crystalline structure of sputtered Sb film was characterized through x-ray diffraction in a grazing incidence configuration (GIDXRD). In the result presented in Figure S1(b) all diffraction peaks can be

assigned to R-3m space group and point group D_{3d}, similar to expected for Sb powder x-ray diffraction pattern.[1] This feature corroborates the growth of high-quality Sb thin film without evidence of secondary phases formation.

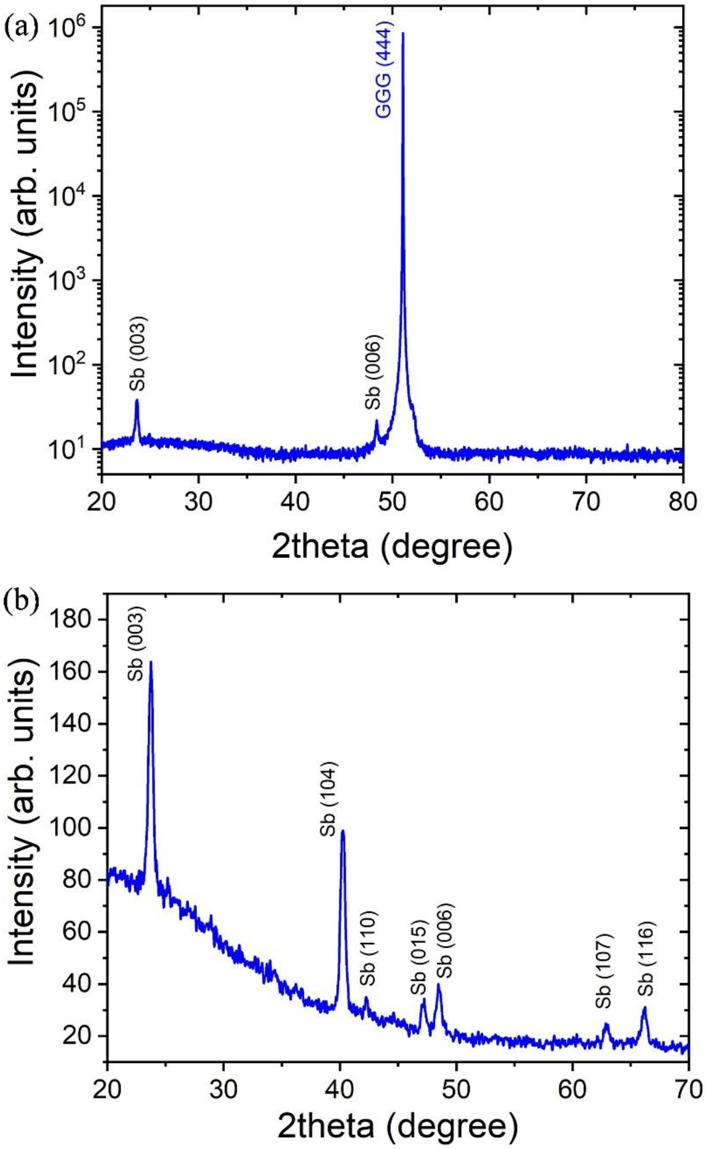

Figure S1. (a) Symmetrical HRXRD measurement and (b) Grazing incidence (GID) measurement of Sb thin film on (111) GGG substrate. All XRD peaks of Sb are indexed as a rhombohedral crystal structure with R-3m space group and point group D_{3d}.

To evaluate the film thickness and surface roughness, we carry out x-ray reflectivity measurements in the sputtered Sb thin films. The result is shown in Figure S2, expressed as a function of the longitudinal momentum transfer vector in the out-of-plane direction $q = 4\pi/\lambda \sin(2\theta/2)$, where λ is the wavelength and 2θ the scattering angle. The observation of an oscillatory pattern is direct evidence of a well-defined interface between Sb film and GGG substrate. The parameters obtained after XRR curve fitting (solid red line) are depicted in the graph. From these results, the total thickness of the film is 86.9 ± 0.5 nm and we can infer that the Sb thin films exhibit a low surface roughness (0.6 ± 0.2 nm) and density of 6.8 ± 0.2 g/cm^3, in good accordance with the expected bulk value.

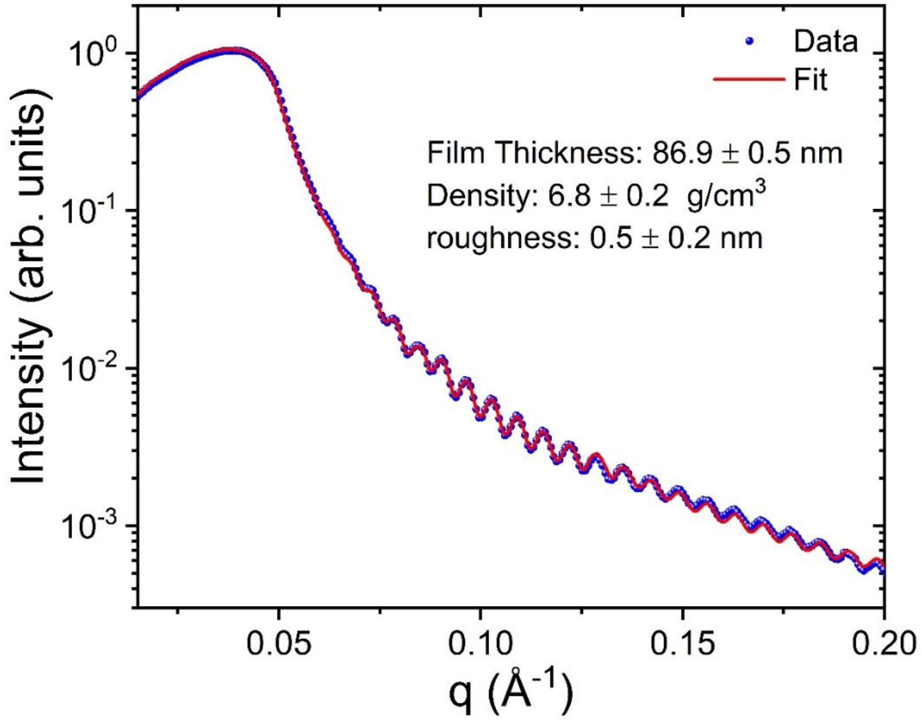

Figure S2. X-ray reflectivity curve (blue dots) of Sb on (111) GGG substrate. The red solid line represents the curve fitting using the Parratt model. *Inset* shows the Sb thin film parameters obtained from fitting the experimental data.

II. Scanning tunneling microscopy (STM) and spectroscopy (STS) characterizations of Sb films

Scanning tunneling microscopy (STM) and spectroscopy (STS) measurements were performed in order to investigate the surface electronic properties of Sb thin film. In a typical STS curve the differential tunneling conductance (dI/dV) is proportional to the local density of states (LDOS) at the STM tip position *(2)* and, therefore, allows us to probe the presence of topologically protected states on the antimony surface.

Figure S3 (left panel) shows a typical STM topographic image of Sb thin film evidencing a flat surface with root-mean-square (rms) roughness of about 0.8 nm in good agreement with our XRR results. Furthermore, the layered structure of sputtered Sb on GGG substrate can be observed due to the presence of well-defined terraces characterized by sharp steps corresponding to the inter Sb bilayer spacing with height of ~ 5.8 Å *(3)* (Figure S3 right panel).

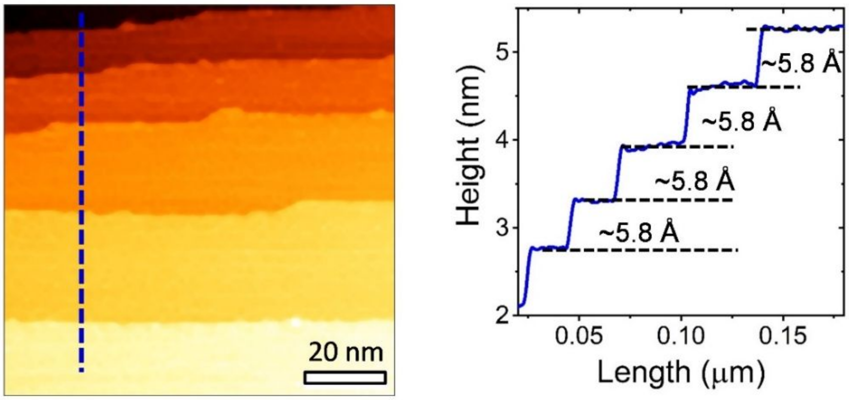

Figure S3. (Left panel) STM topography image of about 100 × 100 nm^2 of the Sb surface at I = 0.3 nA and V = − 0. 5 V. (Right panel) Height profile taken along the blue dashed line indicated in the topography image.

Figure S4 shows a spatially averaged dI/dV spectrum acquired on Sb surface, the region of positive bias voltage denotes the bulk conduction band (BCB) and the region of negative bias voltage denotes the bulk valence band (BVB). The semimetal nature of Sb thin film can be observed through the suppression of LDOS in the vicinity of Fermi level (corresponding to zero of sample bias) indicating an overlapping between the conduction and valence bands.

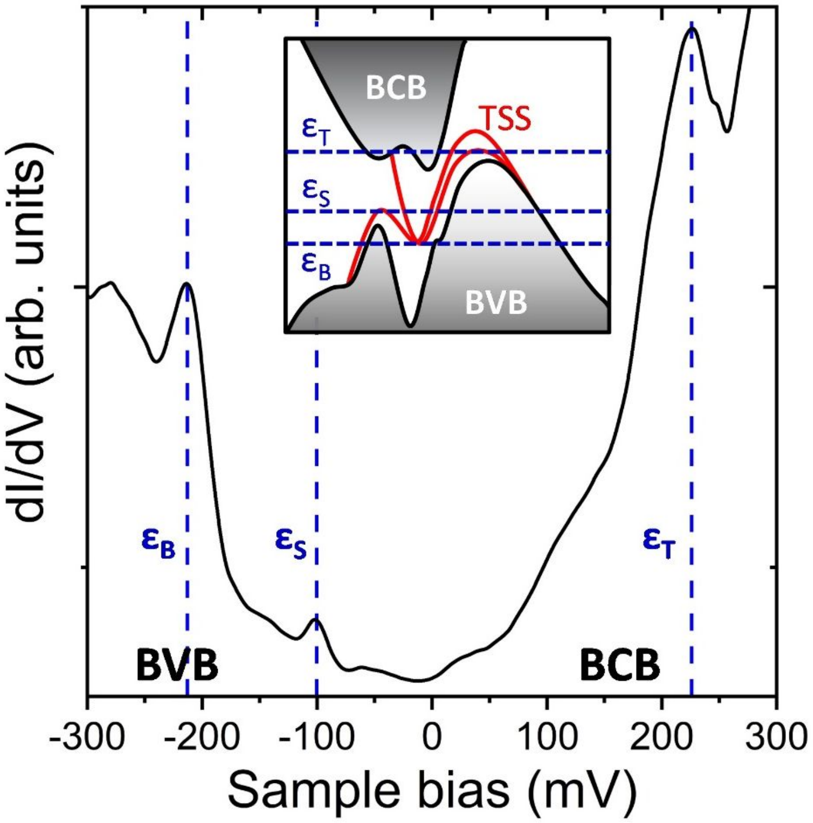

Figure S4. Spatially averaged dI/dV spectrum obtained along the Sb surface. BVB and BCB denotes the regions of bulk valence band and bulk conduction band, respectively. The vertical blue dashed line represents the ε_B, ε_S and ε_T topological surface states features. *Inset* shows a schematic representation of Sb electronic band structure (based on first principles calculations) representing the position of topological surface states with respect BVB and BCB. The zero energy (Sample bias) marks the Fermi level (E_F).

In order to better understand the characteristics of the measured Sb STS spectrum it is necessary to analyze the overall antimony band structure, shown schematically as an inset in figure S4. The Sb semimetallic behavior results in a negative bulk band gap that distorts the topological surface states (TSS), resulting in a single pair of Rashba-split surface states that span the semimetallic gap with a Dirac cone at the zone center. [4,5] The inner cone can persist up to significantly higher

energies, whereas the outer cone must fold down to connect with the bulk valence band. This leads to the appearance of extreme characteristics features within the outer Rashba cone, with one being a saddle point (ε_S) and the other a band edge (ε_T and ε_B). [4-7]

Based on this, the presence of three prominent peaks (vertical blue dashed lines) observed in dI/dV spectra shown in figure S4 can be a signature of TSS in sputtered Sb thin film. Indeed, the dI/dV peaks at − 214 mV and 227 mV are in good agreement with those observed in STS measurement in bulk antimony and can be associated with 2D band edges at ε_B and ε_T, respectively.[4] Meanwhile, the dI/dV peak at − 104 mV usually is associated to the logarithmic singularity associated with the 2D saddle point at ε_S. [4-7]

III. Sample Fabrication

YIG ($Y_3Fe_5O_{12}$) films were grown onto 1-inch diameter GGG($Gd_3Ga_5O_{12}$) substrate via liquid phase epitaxy. Fe_2O_3, Y_2O_3, B_2O_3 and PbO powders were weighed and placed into a platinum crucible which was then inserted into a vertical tubular furnace, the furnace temperature was set to 940°C with a rate of 1°C/min. Once the furnace is up to temperature, the GGG substrate is then placed on a platinum sample holder that slowly descends into the crucible. To grow the YIG film, the GGG substrate dips into the melt inside the crucible, to ensure a uniform film the dipping process is done with the sample rotating at a fixed velocity.

Once the YIG is grown, the film is cut into 3x2 mm^2 samples with a low-speed diamond saw, following the samples are clean in an ultrasonic bath with acetone and isopropyl alcohol, the

sample is then placed inside the sputtering chamber which was pumped down to a base pressure of 2.0×10^{-7} torr or lower; The Sb films were grown by DC sputtering in an argon atmosphere with a working pressure of 2.7×10^{-3} torr. The argon flux to the chamber was 550 sccm and the plasma current was kept fixed at 50 mA. The texture observed in Sb is due to the use of YIG as a substrate. As already mentioned, the YIG films were grown onto a commercial [111] Gadolinium-Gallium Garnets substrate (GGG) via liquid phase epitaxy. Since GGG and YIG presents a very low lattice mismatch (a_{GGG} = 12.385Å, a_{YIG} = 12.376Å, $\frac{\Delta a}{a}$ = 6 x 10^{-4}) the YIG films grows perfectly at the [111] direction. Figure S5 show a diagram of the sample fabrication process.

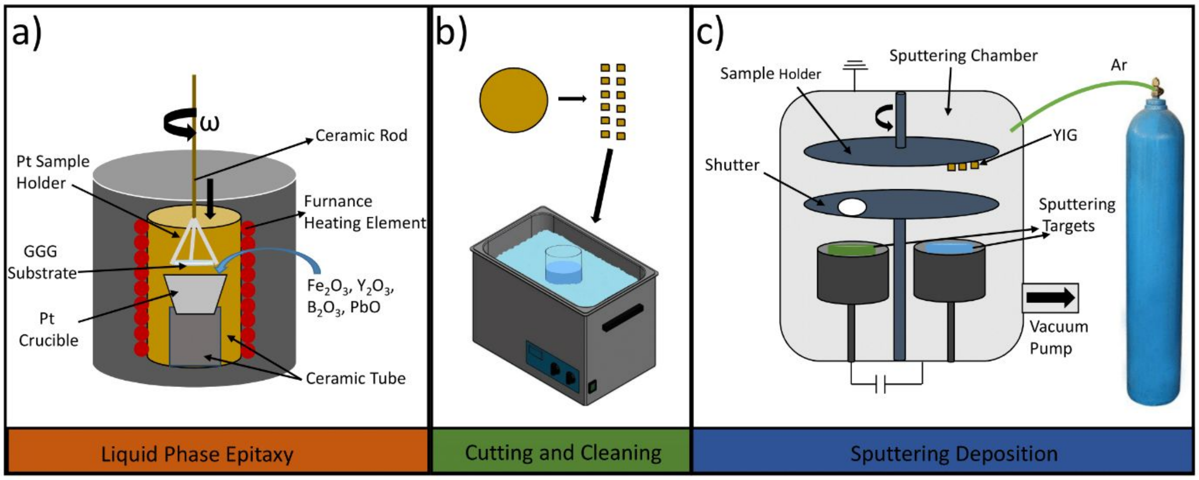

Figure S5. Schematics of the sample fabrication process, in (a) it is presented the liquid phase epitaxy method, in (b) it is show the cutting and cleaning procedures and in (c) it is shown the sputtering deposition process.

IV. Ferromagnetic Resonance (FMR) and Spin pumping measurements

Ferromagnetic Resonance (FMR) measurements were performed in a home-made spectrometer. The sample is mounted on top of a PVC rod and through a small hole in a rectangular microwave cavity operating at TE102, with resonance frequency of 9.42 GHz. The cavity is then placed inside the poles of an electromagnet, the microwave frequency used to excite the FMR condition is directed into the sample via a circulator and wave guides that are connected to the cavity. On the third side of the circulator a RF Schottky diode is placed which allows us to observe the reflected microwave power as function of the applied external field. To have a better noise-to-signal ratio the external field is modulated with two coils in the Helmholtz configuration which are supplied with an AC signal of 1.1 kHz, the diode signal is then sent to a lock-in amplified. In a typical FMR experiment field sweeps are made while observing the reflected microwave power, at the resonance condition the sample will absolve the microwave radiation which will produces a drop in the signal that comes from the diode. Due to the use of a lock-in amplified, the observed signal ends up being the derivative of the reflected microwave power as function of the external field. There are two important properties that can be extract from a FMR specter, the resonance field HR and the linewidth ΔH. The resonance field is related to the anisotropies of the material while the linewidth is related to dissipation mechanism of the film. Figure S6 shows a typical FMR spectra for YIG and YIG/Pt(2nm), where in Fig. S6 (a) we highlighted the two properties we can extract from the typical FMR spectra, note that by adding a thin layer of Pt the linewidth increases from 2.63 Oe to 2.84 Oe.

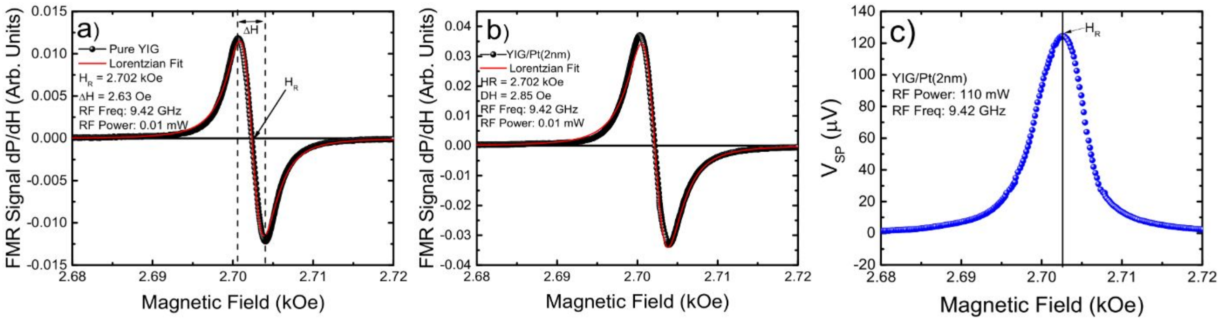

Figure S6. Typical FMR spectra for (a) pure YIG and (b) YIG/Pt(2nm). In (a) it is highlighted the Resonance field H_R and the linewidth ΔH, note that there is an increase in the linewidth because the addition of the Pt(2nm) layer. Spin pumping voltage measured at the edges of the sample is shown in (c).

Spin pumping measurements were performed in the same spectrometer. The magnetization dynamics pumps a pure spin current from the ferromagnetic material into the adjacent layer, since the ferromagnetic material is losing energy in the form of a spin current being injected into the adjacent layer, we observe an increase on the linewidth of the system. Inside the adjacent layer, the spin current can be converted into a charge current via inverse Rashba effect or inverse Spin Hall effect. The electrical signal produced at the resonance condition can be detected via a nanovoltmeter by attaching two electrodes at the edges of the sample with silver paste. Figure S6(c) shows the spin pumping voltage obtained for YIG/Pt(2nm), note that the peak happens at the resonance field. The measured signal is normalized by the sample resistance to remove a geometric dependency of the spin pumping signal with the sample shape and size.

REFERENCES

(1) Barrett, C. S., Cucka, P., and Haefner, K., The crystal structure of antimony at 4.2, 78 and 298˚K, Acta Cryst., 16, 451-453 (1963).

(2) Liu, Y. *et al.* Charging Dirac States at Antiphase Domain Boundaries in the Three-Dimensional Topological insulator Bi_2Se_3, Phys. Rev. Lett. 110, 186804 (2013).

(3) Li, Y. *et al.* Emergent and tunable Topological Surface States in Complementary Sb/Bi_2Te_3 and Bi_2Te_3/Sb Thin film Heterostructures, ACS Nano 16, 9953-9959 (2022).

(4) Soumyanarayanan, A., and Hoffman, J. E., Momentum-resolved STM studies of Rashba-split surface states on the topological semimetal Sb, Journal of Electron Spectroscopy and Related Phenomena 201, 66-73 (2015).

(5) Narayan A., Rungger I., and Sanvito, S. Topological surface states scattering in antimony, Phys Rev. B, 86, 201402 (2012).

(6) Y. Yu, L. She, H. Fu, M. Huang, H. Li, S. Meng, G. Cao. ACS Nano 2014, 8, 11, 11576–11582 (2014).

(7) Yao, G., Luo, Z., Pan, F. Xu, W., Feng, Y.P, and Wang X.-S, Evolution of Topological surface states in Antimony Ultra-Thin Films, Sci. Reports, 3, 2010 (2013).
